# Adverse event reporting of faricimab: a disproportionality analysis of FDA adverse event reporting system (FAERS) database

**DOI:** 10.3389/fphar.2025.1521358

**Published:** 2025-03-12

**Authors:** Chang-Zhu He, Qin Qiu, Song-Jie Lu, Fu-Li Xue, Jun-Qiao Liu, Yu He

**Affiliations:** ^1^ Chengdu University of Traditional Chinese Medicine, Chengdu, Sichuan, China; ^2^ Department of Ophthalmology, Chengdu First People’s Hospital/Chengdu Integrated TCM and Western Medicine Hospital, Chengdu, Sichuan, China

**Keywords:** faricimab, adverse events, FAERS database, disproportionality, pharmacovigilance

## Abstract

**Background:**

Faricimab is the first and only bispecific antibody approved by the U.S. Food and Drug Administration (FDA) for intravitreal injection. Given its increasingly widespread use in retinal vascular diseases, understanding its adverse events (AEs) in real-world settings is crucial. This study employed the FDA Adverse Event Reporting System (FAERS) database to investigate potential safety concerns, with the aim of providing new insights for clinical practice.

**Methods:**

This study conducted a disproportionality analysis of adverse event data from the FAERS database, in which faricimab was identified as the primary suspect, covering the period from the first quarter of 2022 to the second quarter of 2024. To ensure the accuracy and reliability of the study, we employed four types of disproportionality analyses: the reporting odds ratio (ROR), proportional reporting ratio (PRR), multi-item gamma Poisson shrinker (MGPS), and Bayesian confidence propagation neural network (BCPNN). Additionally, the Weibull distribution was utilized to model the risk of adverse events over time.

**Results:**

A total of 2,735 adverse reaction reports, in which faricimab was identified as the primary suspect, were retrieved from the FAERS database. The analysis showed that faricimab-induced AEs occurred across 25 system organ classes (SOCs), with eye disorders meeting the positive threshold for all four algorithms. Significant AEs were mapped to preferred terms (PT), identifying the adverse reactions listed on the drug label: endophthalmitis, elevated intraocular pressure, cataract, retinal pigment epithelial tear, vitreous floaters, retinal vasculitis, retinal artery occlusion, and retinal vein occlusion. In addition to the AEs listed on the drug label, several previously unreported AEs were identified, including blindness, cerebral infarction, retinal hemorrhage, retinal occlusive vasculitis, glaucoma, dry eye, metamorphopsia, and unilateral blindness.

**Conclusion:**

This study provided valuable evidence on the real-world safety of faricimab, suggesting that clinicians should place greater emphasis on monitoring its adverse effects during use.

## 1 Introduction

Age-related macular degeneration (AMD), diabetic macular edema (DME), and retinal vein occlusion-related macular oedema (RVO-MO) are among the leading global causes of blindness and visual impairment (Sharma et al., 2024). AMD is a major cause of visual impairment among the elderly and occurs in two forms: dry (non-neovascular) and wet (neovascular, nAMD). Approximately 90% of severe vision loss associated with AMD is attributable to the wet form (Yang et al., 2022). DMO and RVO-MO are vascular complications linked to systemic diseases such as diabetes and hypertension, DME is marked by fluid accumulation in the macula due to vascular damage, whereas RVO-MO results from retinal vein occlusion, which causes retinal hemorrhages and macular oedema (Panos et al., 2023). Intravitreal injection of anti-vascular endothelial growth factor (anti-VEGF) drugs is the current mainstream treatment approach. Faricimab is the first and currently the only bispecific antibody approved by the U.S. Food and Drug Administration (FDA), administered through intravitreal injection (Liberski et al., 2022). Faricimab binds with high affinity to angiopoietin-2 (Ang-2) and neutralizes VEGF-A, targeting two key pathways involved in the pathology of nAMD and DME, this dual inhibition addresses distinct mechanisms contributing to the progression of these diseases (Zhang et al., 2024). Multiple clinical studies had demonstrated that faricimab is highly effective, leading to improvements in best corrected visual acuity (BCVA), reductions in retinal thickness, and significant anatomical enhancements (Zarbin et al., 2024; Wykoff et al., 2022; Wong et al., 2024). A meta-analysis conducted by Watkins et al. (2023) demonstrated that faricimab achieves greater improvements in BCVA compared to Ranibizumab, while also requiring fewer injections. A phase II clinical trial indicated that the duration of faricimab’s effect may exceed that of existing intravitreal anti-VEGF treatments like ranibizumab, allowing for less frequent dosing regimens (Khanani et al., 2020).

Faricimab exhibits a unique mechanism of action and shows promising potential for expanded clinical applications. Although several studies (Wijesingha et al., 2024; Li et al., 2023) had assessed its safety, real-world safety data remains limited. The FDA Adverse Event Reporting System (FAERS) is one of the largest post-market safety monitoring databases. It collects standardized real-world data to support the FDA’s safety surveillance program for drugs and therapeutic biologics through spontaneous reports submitted by consumers, healthcare professionals, pharmaceutical manufacturers, and other non-medical individuals (Zhao and Tao, 2024). This study performed a retrospective pharmacovigilance analysis using the FAERS database to assess adverse event reports related to faricimab. Signal detection methods were employed to identify potential drug safety signals and offer critical insights into faricimab’s safety profile, providing new evidence for clinical ophthalmologists.

## 2 Materials and methods

### 2.1 Data source and study design

**FIGURE 1:**
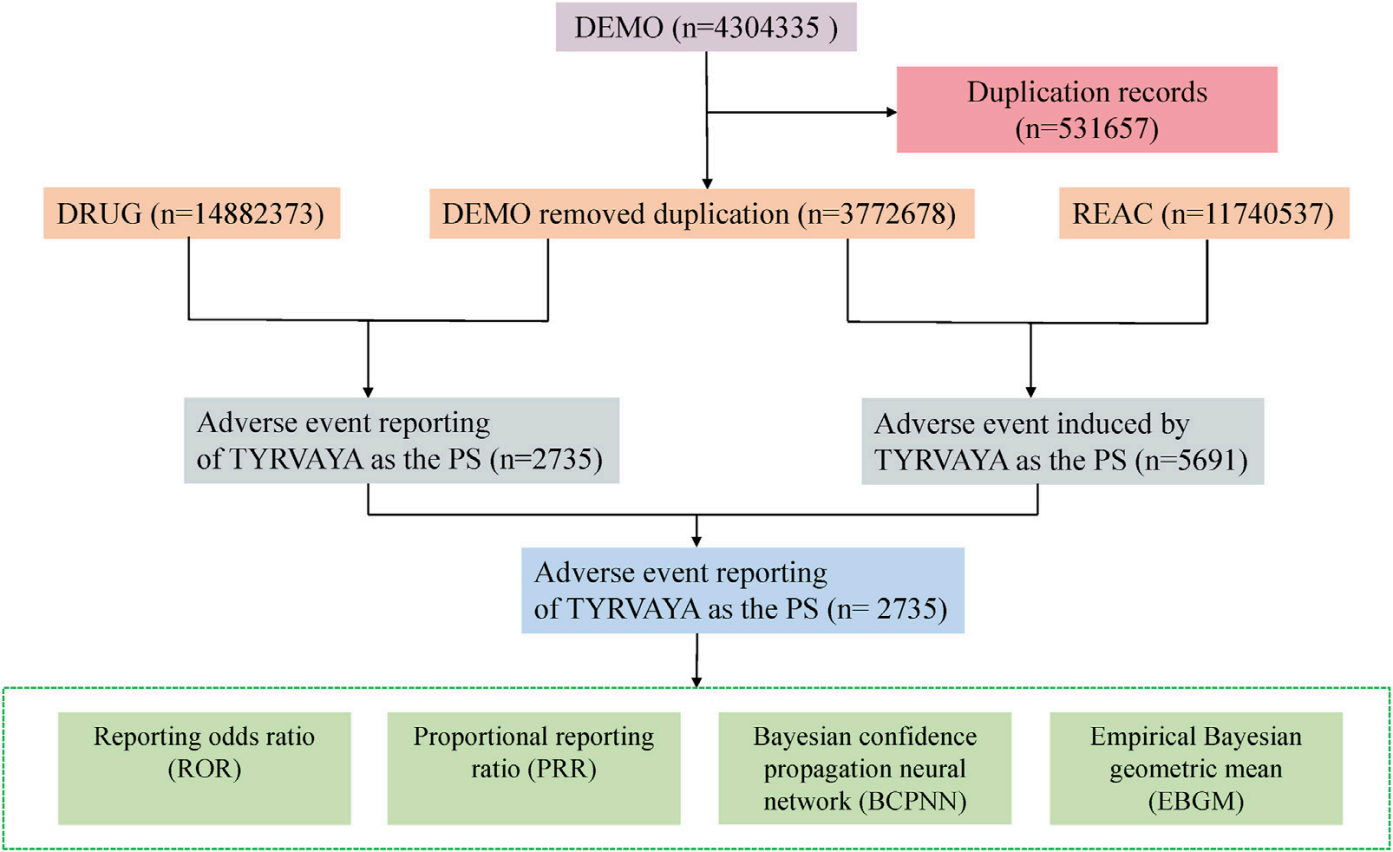
Flowchart of the screening process for faricimab -related adverse events (AEs).

Data for this study were sourced from the FAERS database, a comprehensive pharmacovigilance resource supporting the FDA’s post-marketing surveillance program for approved drugs and therapeutic biologics. Compared to other pharmacovigilance databases, such as the EV database managed by the European Medicines Agency (EMA), which primarily collects, manages, and analyzes individual case safety reports (ICSRs) related to authorized drugs or vaccines in clinical trials within the European Economic Area (EEA) and makes these data publicly available through the EMA website (www.adrreports.eu) (Gozzo et al., 2023; Brancati et al., 2021), or the Italian spontaneous ADR reporting database (Rete Nazionale di Farmacovigilanza, RNF) (Gozzo et al., 2021), the FAERS database exhibits the following characteristics. The FAERS dataset is structured into seven sections: DEMO (patient demographic and administrative information), DRUG (drug-specific details), REAC (coded adverse events), OUTC (patient outcomes), RPSR (report sources), THER (therapy initiation and cessation dates), and INDI (indications for drug use). Reported drugs in FAERS are categorized into four groups: PS (Primary Suspect), SS (Secondary Suspect), C (Concomitant), and I (Interacting). Both the brand name and the generic name are employed to identify records related to faricimab. The search terms include “VABYSMO,” “FARICIMAB,” “Vascular Endothelial Growth Factor Inhibitors Faricimab,” and “Blinded Faricimab.” In this study, we focused exclusively on data that designated faricimab as a PS. Adverse events (AEs) and medication errors are coded using terminology from the Medical Dictionary for Regulatory Activities (MedDRA), a comprehensive and detailed standard developed by the International Council for Harmonisation of Technical Requirements for Pharmaceuticals for Human Use (ICH). To address duplicate reports, we adopted the methodology recommended by the FDA. From the DEMO table, we extracted the PRIMARYID, CASEID, and FDA_DT fields, selecting entries with the maximum FDA_DT value according to FDA guidelines to ensure that we retained the most recent report for each CASEID. In cases where CASEID and FDA_DT are identical, the report with the highest PRIMARYID was retained. The detailed flowchart of the research design can be found in Figure 1.

### 2.2 Statistical analysis

Disproportionality analysis was employed to characterize the features of adverse event reports associated with faricimab. This analysis typically comprises two components: frequentist statistics and Bayesian statistics. Frequentist Statistics includes the reporting odds ratio (ROR) and proportional reporting ratio (PRR) (Rothman et al., 2004; Evans et al., 2001), while Bayesian Statistics encompasses the multi-item gamma Poisson shrinker (MGPS) and Bayesian confidence propagation neural network (BCPNN) (Bate et al., 1998; Szarfman et al., 2004). In this study, AEs that met the positive thresholds for all four methods were classified as adverse reactions. Combining the ROR, PRR, MGPS, and BCPNN algorithms leveraged the strengths of multiple approaches and mitigated potential bias associated with reliance on a single algorithm. The interval between the occurrence of AEs recorded in the DEMO file and the initiation of faricimab treatment documented in the THER file was defined as the onset time of faricimab-related AEs. Temporal changes in the incidence of AEs were modeled using the Weibull distribution. All analyses were conducted using R software version 4.3.0. The 2 × 2 contingency table used in the descriptive analysis is provided in Table 1, and the specific algorithms and positive threshold criteria for the four methods are detailed in Table 2.

**TABLE 1:** Two-by-two contingency table for disproportionality analyses.

	Target AEs	Other AEs	Total
	a	b	a+b
Other drugs	c	d	c + d
Total	a+c	b + d	a+b + c + d

Abbreviation: AEs, adverse events; a, number of reports containing both the target drug and target adverse drug reaction; b, number of reports containing other adverse drug reaction of the target drug; c, number of reports containing the target adverse drug reaction of other drugs; d, number of reports containing other drugs and other adverse drug reactions.

**TABLE 2:** Four major algorithms used for signal detection.

Algorithms	Equation	Criteria
ROR	ROR = ad/b/c	lower limit of 95% CI > 1, N ≥ 3
	95%CI = e^ln(ROR)±1.96(1/a+1/b+1/c+1/d)^0.5^	
PRR	PRR = a (c + d)/c/(a+b)	PRR≥2, χ^2^ ≥ 4, N ≥ 3
	χ^2^ = [(ad-bc)^2](a+b + c + d)/[(a+b) (c + d) (a+c) (b + d)]	
BCPNN	IC = log_2_a (a+b + c + d) (a+c) (a+b)	IC025 > 0
	95%CI = E (IC) ± 2V(IC)^0.5	
MGPS	EBGM = a (a+b + c + d)/(a+c)/(a+b)	EBGM05 > 2
	95%CI = e^ln(EBGM)±1.96(1/a+1/b+1/c+1/d)^0.5^	

Abbreviation: a, number of reports containing both the target drug and target adverse drug reaction; b, number of reports containing other adverse drug reaction of the target drug; c, number of reports containing the target adverse drug reaction of other drugs; d, number of reports containing other drugs and other adverse drug reactions. 95%CI, 95% confidence interval; N, the number of reports; χ2, chi-squared; IC, information component; IC025, the lower limit of 95% CI of the IC; E (IC), the IC expectations; V(IC), the variance of IC; EBGM, empirical Bayesian geometric mean; EBGM05, the lower limit of 95% CI of EBGM.

## 3 Results

### 3.1 Basic population characteristics

Between the first quarter of 2022 and the second quarter of 2024, the FAERS database received a total of 4,304,335 reports. Following data deduplication and screening, 5,691 adverse reaction reports involving 2,735 patients were identified, with faricimab designated as the PS drug. Table 3 demonstrated the basic population characteristics with faricimab-related AEs. The proportion of female patients (39.6%) exceeded that of male patients (35.5%), with the gender of the remaining patients unspecified. Regarding age distribution, patients aged 65–85 represented the largest group (32.1%), followed by those aged 18–65 (17%). The United States accounted for more than half (55.6%) of reported cases, with Japan, Canada, the United Kingdom, and India comprising the remainder of the top five reporting countries. In terms of reporting sources, clinicians contributed the largest proportion of reports (61.3%), followed by consumers, health professionals, and pharmacists. Since its launch in 2022, the number of reported AEs has shown a steady annual increase, peaking in 2024 with 67.7% of the total reports.

**TABLE 3:** Clinical characteristics of faricimab adverse event reports from the FAERS database (Q1 2022 – Q2 2024).

Characteristics	Case numbers	Case proportion (%)
Number of events	2735	
Gender		
Male	970	35.5
Female	1083	39.6
Miss	682	24.9
Age		
<18	56	2.0
18–65	464	17.0
65–85	877	32.1
>85	181	6.6
Miss	1157	42.3
Reporting countries (Top 5)		
United States	1521	55.6
Japan	211	7.7
Canada	135	4.9
United Kingdom	127	4.6
India	126	4.6
Reporter		
Physician	1677	61.3
Consumer	809	29.6
Health professionals	193	7.1
Pharmacist	50	1.8
Miss	6	0.2
Reporting year		
2022 2023 2024	142 740 1853	5.2 27.1 67.7

### 3.2 Signal detection of faricimab at the system organ class (SOC) level

**FIGURE 2:**
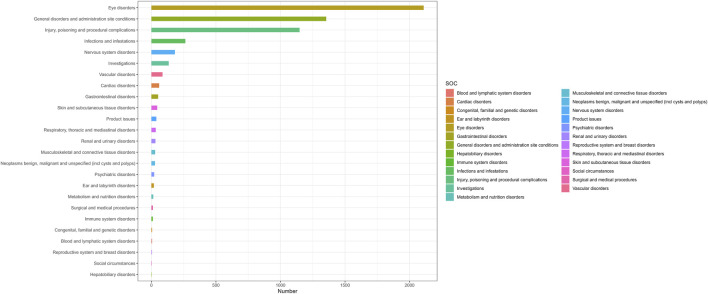
Proportion of adverse events by System Organ Classes (SOC) for faricimab.

The results of faricimab’s AE reports at the SOC level are presented in Table 4. The proportion of SOC reported in faricimab-related AEs can be found in Figure 2. The final analysis revealed that adverse reactions associated with faricimab spanned 25 SOCs. Ranked by the number of reported cases, the top three SOCs were Eye Disorders (n = 2109, 37.1%), General Disorders and Administration Site Conditions (n = 1354, 23.8%), and Injury, Poisoning, and Procedural Complications (n = 1148, 20.1%). Eye Disorders (ROR = 29.56, PRR = 18.97, EBGM = 18.81, IC = 4.23) demonstrated a strong positive signal across all four algorithms, aligning with descriptions in the faricimab drug label, which suggests high data reliability. Additionally, General Disorders and Administration Site Conditions (ROR = 1.43) met the positive threshold only in the ROR algorithm, while displaying negative signals in the other algorithms.

**TABLE 4:** Signal strength of faricimab AEs across System Organ Classes (SOC) in the FAERS database.

System Organ Class (SOC)	Case numbers	ROR (95%Cl)	PRR (χ2)	EBGM(EBGM05)	IC(IC025)
Vascular disorders	86	0.83 (0.67–1.02)	0.83 (3.09)	0.83 (0.69)	−0.27 (−1.94)
Neoplasms benign, malignant and unspecified (incl cysts and polyps)	28	0.15 (0.11–0.22)	0.16 (129.63)	0.16 (0.12)	−2.66 (−4.33)
Eye disorders*	2,109	29.56 (28.01–31.2)	18.97 (36,299.01)	18.81 (17.98)	4.23 (2.57)
Cardiac disorders	60	0.56 (0.44–0.72)	0.57 (20.33)	0.57 (0.46)	−0.82 (−2.49)
Infections and infestations	263	0.75 (0.66–0.85)	0.76 (20.64)	0.76 (0.69)	−0.39 (−2.06)
Nervous system disorders	182	0.43 (0.37–0.5)	0.45 (133.87)	0.45 (0.4)	−1.16 (−2.83)
Investigations	134	0.39 (0.33–0.46)	0.4 (127.29)	0.4 (0.35)	−1.32 (−2.98)
Renal and urinary disorders	31	0.33 (0.23–0.47)	0.33 (41.69)	0.33 (0.25)	−1.58 (−3.25)
Gastrointestinal disorders	52	0.11 (0.08–0.14)	0.12 (379.67)	0.12 (0.09)	−3.11 (−4.77)
General disorders and administration site conditions	1,354	1.43 (1.34–1.52)	1.32 (131.38)	1.32 (1.26)	0.41 (−1.26)
Blood and lymphatic system disorders	5	0.05 (0.02–0.12)	0.05 (91.97)	0.05 (0.02)	−4.32 (−5.99)
Respiratory, thoracic and mediastinal disorders	34	0.13 (0.09–0.18)	0.13 (202.24)	0.13 (0.1)	−2.92 (−4.58)
Metabolism and nutrition disorders	15	0.14 (0.08–0.23)	0.14 (81.19)	0.14 (0.09)	−2.84 (−4.51)
Injury, poisoning and procedural complications	1,148	1.66 (1.55–1.77)	1.53 (239.65)	1.53 (1.45)	0.61 (−1.06)
Ear and labyrinth disorders	19	0.82 (0.52–1.29)	0.82 (0.73)	0.82 (0.56)	−0.28 (−1.95)
Product issues	38	0.33 (0.24–0.45)	0.33 (51.81)	0.33 (0.26)	−1.59 (−3.25)
Musculoskeletal and connective tissue disorders	29	0.09 (0.06–0.13)	0.1 (255.46)	0.1 (0.07)	−3.36 (−5.02)
Surgical and medical procedures	12	0.13 (0.07–0.23)	0.13 (70.29)	0.13 (0.08)	−2.93 (−4.6)
Immune system disorders	11	0.17 (0.09–0.31)	0.17 (44.13)	0.17 (0.11)	−2.53 (−4.2)
Skin and subcutaneous tissue disorders	45	0.15 (0.11–0.2)	0.15 (219.81)	0.15 (0.12)	−2.7 (−4.36)
Psychiatric disorders	22	0.07 (0.05–0.11)	0.08 (264.07)	0.08 (0.05)	−3.73 (−5.4)
Social circumstances	3	0.11 (0.04–0.35)	0.11 (20.58)	0.11 (0.04)	−3.12 (−4.79)
Hepatobiliary disorders	2	0.04 (0.01–0.17)	0.04 (43.77)	0.04 (0.01)	−4.56 (−6.23)
Congenital, familial and genetic disorders	5	0.35 (0.15–0.84)	0.35 (6)	0.35 (0.17)	−1.51 (−3.18)
Reproductive system and breast disorders	4	0.12 (0.05–0.33)	0.12 (25.01)	0.12 (0.05)	−3.02 (−4.68)

Abbreviation: Asterisks (*) indicate statistically significant signals in four algorithms; ROR, reporting odds ratio; PRR, proportional reporting ratio; EBGM, empirical Bayesian geometric mean; EBGM05, the lower limit of the 95% CI of EBGM; IC, information component; IC025, the lower limit of the 95% CI of the IC; CI, confidence interval; AEs, adverse events.

### 3.3 Signal detection of faricimab at the preferred terms (PT) level

**FIGURE 3:**
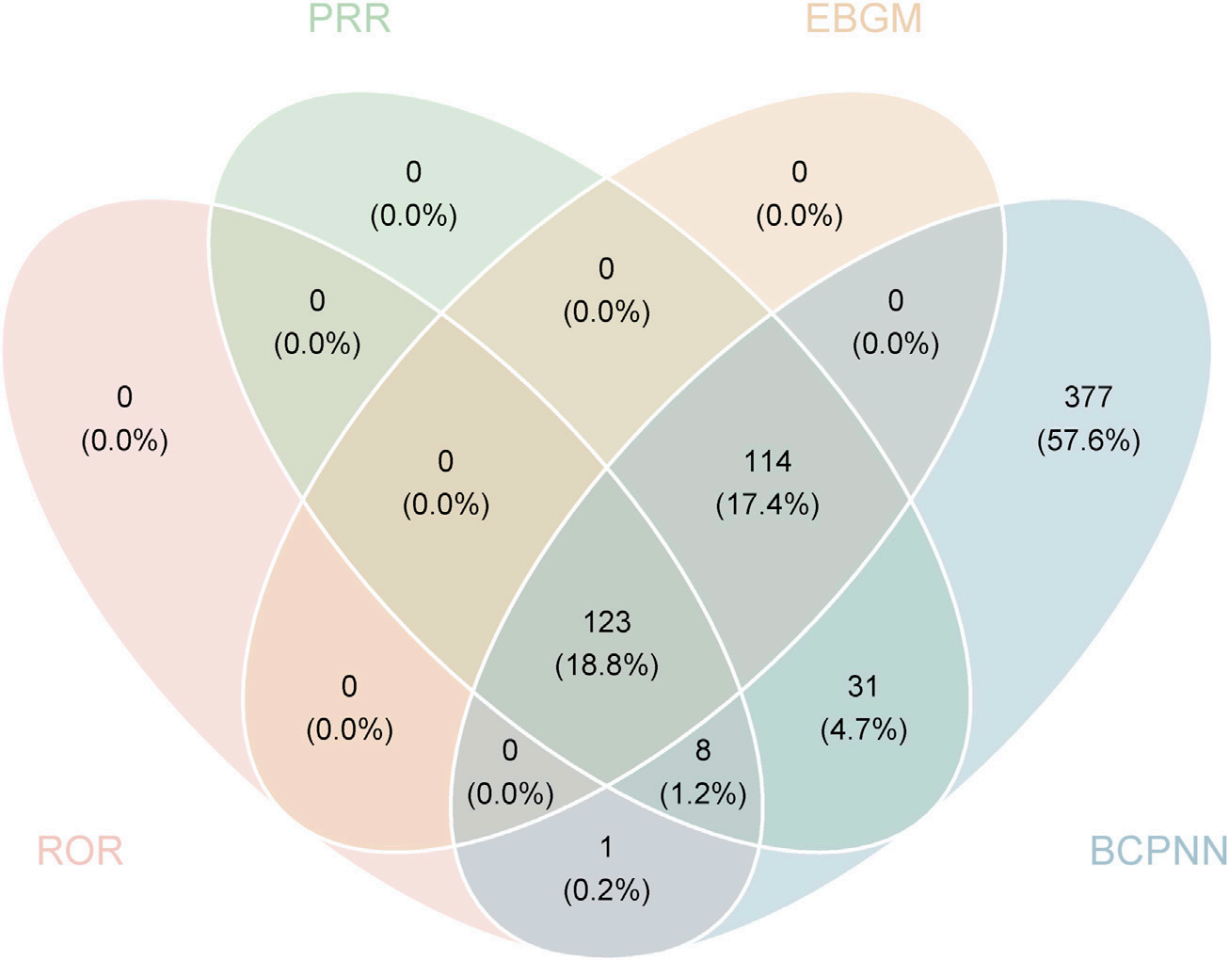
Venn diagram of preferred term (PT) signals meeting the criteria of four algorithms.

The final results showed that 123 PTs met the positive criteria across all four algorithms. Table 5 presents the top 50 AEs associated with faricimab at the PT level. The Venn diagram in Figure 3 visually illustrated the AEs that met the positive threshold of all four algorithms at the PT level. Among the top 50 most common AEs, several events were identified that aligned with those listed on the drug label, including endophthalmitis (Including AEs classified as part of endophthalmitis), elevated intraocular pressure, cataract, retinal pigment epithelial tear, vitreous floaters, retinal vasculitis, retinal artery occlusion, and retinal vein occlusion. Additionally, several noteworthy AEs not included on the drug label were identified, such as blindness, cerebral infarction, retinal hemorrhage, retinal occlusive vasculitis, glaucoma, dry eye, metamorphopsia, and unilateral blindness.

**TABLE 5:** Top 50 frequency of adverse events at the PT level for faricimab.

PT	Case numbers	ROR (95%Cl)	PRR (χ^2^)	EBGM(EBGM05)	IC(IC025)
Visual impairment*	176	15.67 (13.48–18.22)	15.21 (2324.96)	15.11 (13.32)	3.92 (2.25)
Uveitis*	134	76.62 (64.36–91.22)	74.84 (9,423.47)	72.25 (62.45)	6.17 (4.51)
Endophthalmitis*	115	245.44 (201.97–298.26)	240.5 (24,565.11)	215.48 (183.05)	7.75 (6.08)
Vitrifies*	104	723.29 (577.92–905.24)	710.09 (54,779.8)	528.45 (437.99)	9.05 (7.37)
Vitreous floaters*	99	120.77 (98.46–148.14)	118.68 (10,925.58)	112.28 (94.64)	6.81 (5.14)
Vision blurred*	93	8.77 (7.14–10.77)	8.64 (627.07)	8.61 (7.25)	3.11 (1.44)
Blindness*	91	26.69 (21.67–32.88)	26.28 (2186.48)	25.96 (21.81)	4.7 (3.03)
Iridocyclitis*	77	286.38 (225.36–363.91)	282.52 (18,998.23)	248.59 (203.43)	7.96 (6.29)
Intraocular pressure increased*	68	89.47 (70.09–114.2)	88.41 (5,635.75)	84.82 (69.14)	6.41 (4.74)
Eye pain*	67	13.61 (10.69–17.33)	13.46 (768.47)	13.38 (10.93)	3.74 (2.08)
Retinal haemorrhage*	58	148.24 (113.43–193.74)	146.74 (7,838.33)	137.06 (109.56)	7.1 (5.43)
Iritis*	43	182.69 (133.61–249.8)	181.32 (7,088.11)	166.75 (128.34)	7.38 (5.71)
Cataract*	41	7.25 (5.33–9.86)	7.21 (218.57)	7.18 (5.55)	2.84 (1.18)
Retinal pigment epithelial tear*	36	2334.51 (1449.03–3,761.1)	2319.75 (39,265.66)	1092.18 (732.81)	10.09 (8.39)
Pain	33	0.47 (0.34–0.67)	0.48 (19.05)	0.48 (0.36)	−1.06 (−2.73)
Anterior chamber inflammation*	32	850.73 (563.3–1284.83)	845.95 (19,150.18)	600.15 (425.05)	9.23 (7.54)
Keratic precipitates*	31	522.53 (352.1–775.45)	519.69 (12,818.12)	415.28 (298.46)	8.7 (7.02)
Retinal vasculitis*	28	227.53 (153.88–336.42)	226.42 (5,662.19)	204.11 (147.15)	7.67 (6)
Retinal occlusive vasculitis*	25	809.02 (509.19–1285.39)	805.47 (14,444.52)	579.49 (393.37)	9.18 (7.48)
Vitreous haemorrhage*	23	117.57 (77.18–179.11)	117.1 (2505.34)	110.86 (77.95)	6.79 (5.12)
Headache	23	0.44 (0.29–0.66)	0.44 (16.25)	0.44 (0.31)	−1.17 (−2.84)
Ocular hyperaemia*	22	5.12 (3.36–7.78)	5.1 (72.41)	5.09 (3.58)	2.35 (0.68)
Corneal oedema*	20	120.3 (76.59–188.97)	119.88 (2228.36)	113.35 (77.68)	6.82 (5.15)
Dry eye*	20	3.54 (2.28–5.49)	3.53 (36.28)	3.53 (2.44)	1.82 (0.15)
Hypopyon*	19	354.13 (217.54–576.51)	352.96 (5,693.68)	301.52 (200.55)	8.24 (6.55)
Eye irritation*	19	3.97 (2.53–6.24)	3.96 (42.05)	3.96 (2.71)	1.98 (0.32)
Lacrimation increased*	18	6.06 (3.81–9.63)	6.04 (75.59)	6.03 (4.09)	2.59 (0.93)
Cerebrovascular accident*	17	1.71 (1.06–2.76)	1.71 (5.04)	1.71 (1.15)	0.78 (−0.89)
Photophobia*	17	10.14 (6.29–16.34)	10.11 (138.97)	10.07 (6.75)	3.33 (1.66)
Cerebral infarction*	17	10.83 (6.72–17.45)	10.8 (150.41)	10.75 (7.21)	3.43 (1.76)
Blindness unilateral*	16	15.57 (9.52–25.49)	15.53 (215.97)	15.42 (10.21)	3.95 (2.28)
Retinal artery occlusion*	15	69.22 (41.35–115.86)	69.04 (973.27)	66.84 (43.43)	6.06 (4.39)
Fall	15	0.55 (0.33–0.91)	0.55 (5.62)	0.55 (0.36)	−0.87 (−2.53)
Dizziness	15	0.38 (0.23–0.63)	0.38 (15.41)	0.38 (0.25)	−1.4 (−3.07)
Idiopathic orbital inflammation*	14	459.35 (257.27–820.17)	458.22 (5,225.88)	375.09 (230.93)	8.55 (6.85)
Retinal vein occlusion*	13	68.36 (39.32–118.86)	68.21 (833.37)	66.06 (41.58)	6.05 (4.37)
Anterior chamber cell*	13	189.2 (107.16–334.08)	188.77 (2224.52)	173.03 (107.53)	7.43 (5.75)
Glaucoma*	13	8.53 (4.95–14.72)	8.52 (85.89)	8.48 (5.38)	3.08 (1.42)
Chorioretinitis*	13	197.55 (111.77–349.18)	197.1 (2315.14)	179.99 (111.76)	7.49 (5.81)
Myocardial infarction	12	1.61 (0.91–2.83)	1.6 (2.73)	1.6 (1)	0.68 (−0.98)
Vitreous opacities*	12	119.79 (66.9–214.49)	119.54 (1333.27)	113.04 (69.43)	6.82 (5.14)
Eye discharge*	12	11.91 (6.75–21.02)	11.89 (119.03)	11.83 (7.35)	3.56 (1.9)
Eye swelling*	12	4.33 (2.46–7.63)	4.32 (30.61)	4.32 (2.69)	2.11 (0.44)
Non-infectious endophthalmitis*	12	189.28 (104.74–342.05)	188.89 (2054.52)	173.12 (105.52)	7.44 (5.75)
Hypertension	11	0.58 (0.32–1.04)	0.58 (3.41)	0.58 (0.35)	−0.79 (−2.46)
Metamorphopsia*	11	82.34 (45.04–150.51)	82.18 (848.34)	79.07 (47.73)	6.31 (4.63)
Rash	11	0.28 (0.15–0.5)	0.28 (20.6)	0.28 (0.17)	−1.84 (−3.51)
Pneumonia	10	0.36 (0.19–0.66)	0.36 (11.62)	0.36 (0.21)	−1.48 (−3.15)
Disease progression	10	0.72 (0.39–1.33)	0.72 (1.12)	0.72 (0.43)	−0.48 (−2.15)
Fatigue	10	0.13 (0.07–0.24)	0.13 (58.52)	0.13 (0.08)	−2.93 (−4.6)

Abbreviation: Asterisks (*) indicate statistically significant signals in four algorithms; PT, preferred term; ROR, reporting odds ratio; PRR, proportional reporting ratio; EBGM, empirical Bayesian geometric mean; EBGM05, the lower limit of the 95% CI of EBGM; IC, information component; IC025, the lower limit of the 95% CI of the IC; CI, confidence interval.

### 3.4 Time-to-onset analysis and weibull distribution analysis

**FIGURE 4:**
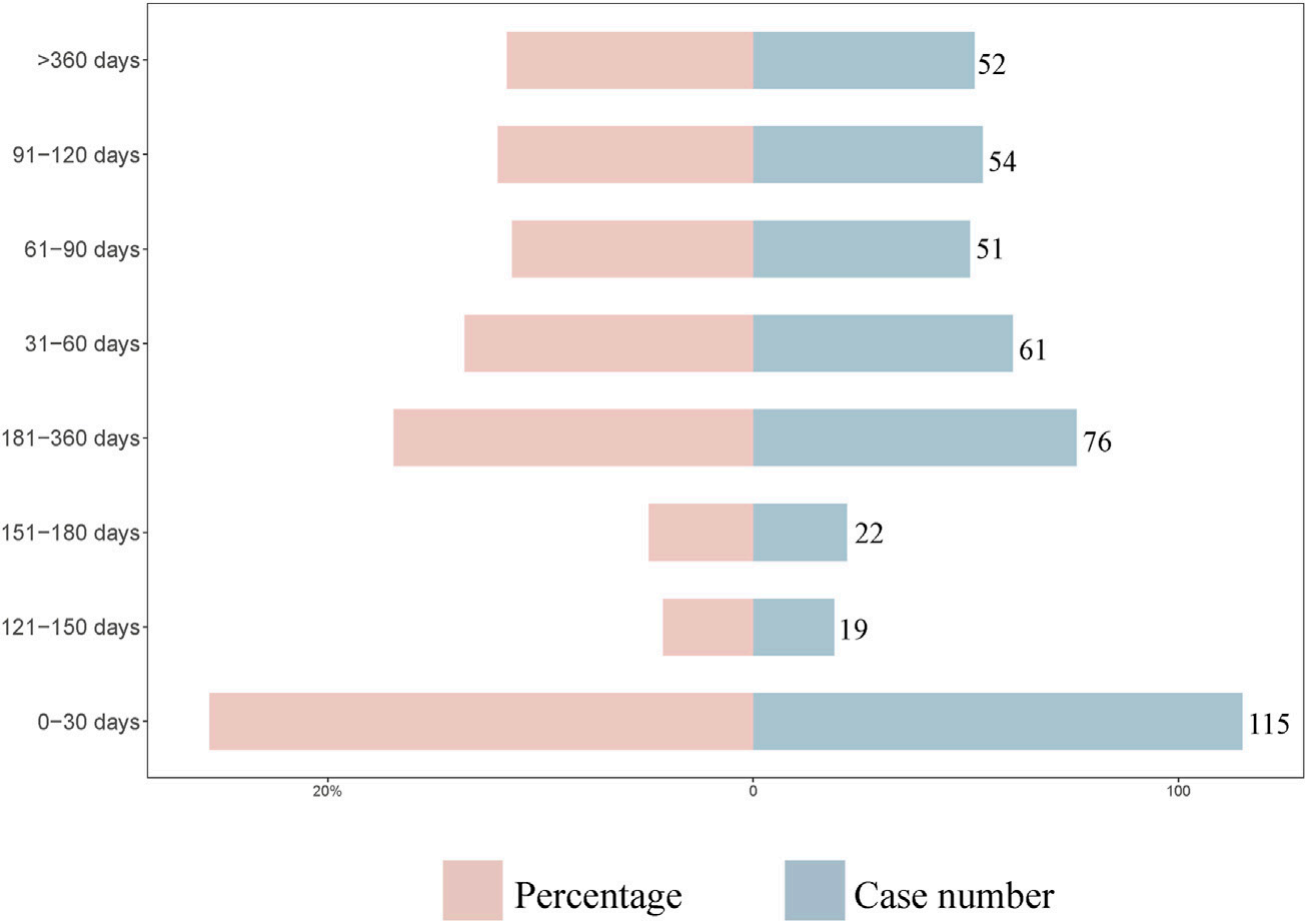
Time to onset of faricimab -related adverse events (AEs).

**FIGURE 5:**
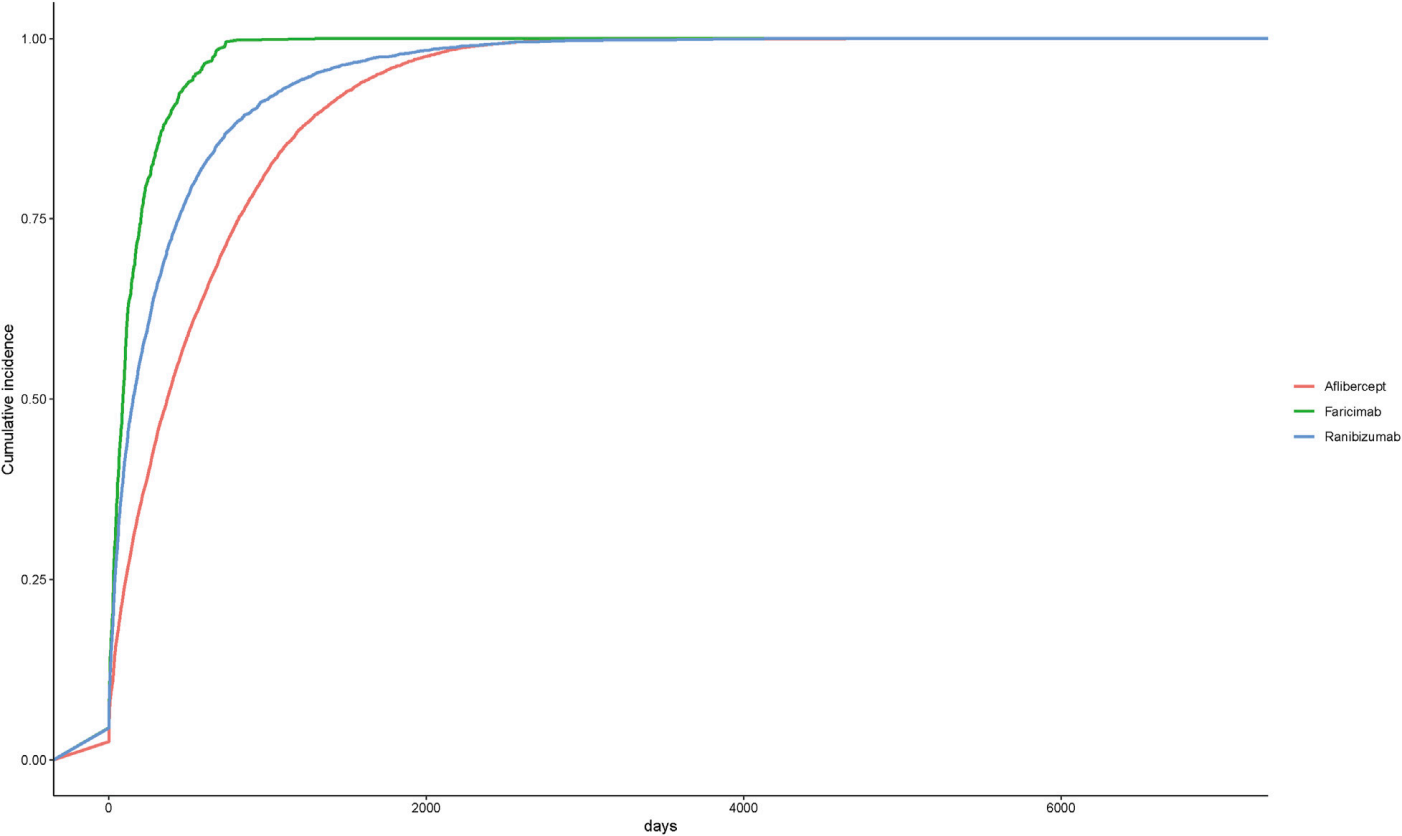
Cumulative incidence of adverse events related to three different anti-VEGF drugs over time.

A total of 450 AEs were associated with onset times, predominantly occurring within the first month. The distribution of onset times for these AEs is shown in Figure 4. Analysis using the Weibull distribution revealed an early failure mode, with detailed parameters presented in Table 6. Additionally, the cumulative incidence curve of AEs is depicted in Figure 5.

**TABLE 6:** Time to onset of faricimab-associated adverse events and Weibull distribution analysis.

Drug	TTO (days)		Weibull distribution		
	Case reports	Median(d) (IQR)	Scale parameter: α (95%CI)	Shape parameter: β (95%CI)	Type
faricimab	450	90 (30–203)	133.72 (117.49–149.95)	0.80 (0.74–0.85)	Early failure

Abbreviation: TTO, time to onset; CI, confidence interval; IQR, interquartile range.

### 3.5 The comparison of ocular and non-ocular AEs between three different anti-VEGF drugs

In order to further explore the safety differences between faricimab and other anti-VEGF drugs, this study also utilized the FAERS database to analyze the adverse reactions of ranibizumab and aflibercept and directly compared them with faricimab. The results revealed that all three anti-VEGF drugs exhibited several similar AEs, including endophthalmitis, retinal vein occlusion, retinal artery occlusion, glaucoma, dry eye syndrome, and additional AEs not mentioned in the drug’s prescribing information. Additionally, among the ocular-related AEs, retinal occlusive vasculitis exhibited positive signals exclusively in aflibercept and faricimab, macular ischemia showed a positive signal only in ranibizumab, and eye edema, retinal depigmentation, and ulcerative keratitis demonstrated positive signals exclusively in ranibizumab and aflibercept. Among the non-ocular AEs, cerebral infarction was a common event across all three drugs, whereas myocardial infarction, transient ischemic attack, and deafness exhibited positive signals solely in ranibizumab. The detailed adverse reactions can be found in Tables 7, 8.

**TABLE 7:** The comparison of ocular adverse reactions among three different anti-VEGF drugs.

PT	Ranibizumab		Aflibercept		Faricimab	
	N	ROR (95%CI)	N	ROR (95%CI)	N	ROR (95%CI)
Visual impairment	1187	11.53 (10.88–12.22)	1396	15.87 (15.04–16.75)	176	15.67 (13.48–18.22)
Endophthalmitis	600	133.26 (122.33–145.17)	1092	353.35 (329.57–378.84)	115	245.44 (201.97–298.26)
Uveitis	106	9.47 (7.82–11.46)	222	22.7 (19.87–25.94)	134	76.62 (64.36–91.22)
Blindness	799	24.16 (22.51–25.93)	1072	41.42 (38.93–44.06)	91	26.69 (21.67–32.88)
Cataract	532	10.81 (9.92–11.78)	402	9.69 (8.78–10.69)	41	7.25 (5.33–9.86)
Retinal haemorrhage	636	123.01 (113.23–133.62)	394	105.37 (94.93–116.96)	58	148.24 (113.43–193.74)
Vitreous floaters	464	61.17 (55.68–67.21)	517	81 (74.01–88.64)	99	120.77 (98.46–148.14)
Vitreous haemorrhage	361	181.54 (162.23–203.15)	186	116.51 (100.04–135.67)	23	117.57 (77.18–179.11)
Retinal pigment epithelial tear	277	789.28 (674.09–924.16)	61	129.65 (99.24–169.38)	36	2334.51 (1449.03–3,761.1)
Intraocular pressure increased	301	27.34 (24.38–30.67)	569	71.78 (65.89–78.19)	68	89.47 (70.09–114.2)
Glaucoma	211	12.89 (11.25–14.77)	128	9.62 (8.08–11.46)	13	8.53 (4.95–14.72)
Dry eye	156	4.16 (3.55–4.86)	85	2.54 (2.05–3.14)	20	3.54 (2.28–5.49)
Retinal vein occlusion	160	75.56 (64.33–88.75)	57	35.44 (27.2–46.16)	13	68.36 (39.32–118.86)
Retinal artery occlusion	88	45.29 (36.57–56.09)	114	74.63 (61.67–90.31)	15	69.22 (41.35–115.86)
Retinal occlusive vasculitis*	2	17.45 (4.31–70.64)	5	43.89 (17.91–107.53)	25	809.02 (509.19–1285.39)
Iridocyclitis	46	18.57 (13.87–24.86)	75	35.84 (28.46–45.14)	77	286.38 (225.36–363.91)
Eye discharge	129	15.66 (13.15–18.63)	61	8.46 (6.58–10.89)	12	11.91 (6.75–21.02)
Punctate keratitis*	11	15.16 (8.36–27.5)	4	6.53 (2.44–17.45)	NR	NR
Keratic precipitates	12	36.07 (20.28–64.17)	21	63.56 (40.88–98.85)	31	522.53 (352.1–775.45)
Lacrimation increased	230	9.42 (8.27–10.73)	201	9.5 (8.27–10.92)	18	6.06 (3.81–9.63)
Macular ischaemia*	26	269.56 (174.75–415.79)	1	12.49 (1.74–89.79)	NR	NR
Subretinal fibrosis	57	502.35 (365.31–690.79)	7	77.5 (35.89–167.35)	6	728.53 (287.13–1848.48)
Eye oedema*	68	33.32 (26.16–42.43)	46	29.49 (21.99–39.55)	2	10.52 (2.62–42.24)
Retinal depigmentation*	41	193.45 (138.43–270.34)	10	46.39 (423.93)	1	44.83 (6.18–325.16)
Ulcerative keratitis*	23	9.87 (6.55–14.89)	18	10.18 (6.4–16.2)	1	4.32 (0.61–30.76)

Abbreviation: The asterisk (*) indicates that this adverse reaction is not a common adverse reaction shared by all three drugs. PT, preferred term; ROR, reporting odds ratio; NR, not report; N, number of target adverse events of target drug; CI, confidence interval.

**TABLE 8:** The comparison of non-ocular adverse reactions among three different anti-VEGF drugs.

PT	Ranibizumab		Aflibercept		Faricimab	
	N	ROR (95%CI)	N	ROR (95%CI)	N	ROR (95%CI)
Headache	360	0.66 (0.59–0.73)	266	0.59 (0.53–0.67)	23	0.44 (0.29–0.66)
Cerebral infarction	250	12.18 (10.75–13.81)	60	3.78 (2.93–4.87)	17	10.83 (6.72–17.45)
Dizziness	218	0.51 (0.45–0.58)	111	0.32 (0.27–0.39)	15	0.38 (0.23–0.63)
Myocardial infarction*	606	4.14 (3.82–4.49)	179	1.89 (1.63–2.19)	12	1.61 (0.91–2.83)
Hypertension	300	1.67 (1.49–1.87)	144	1.01 (0.85–1.18)	11	0.58 (0.32–1.04)
Rash	74	0.2 (0.16–0.25)	29	0.09 (0.07–0.13)	11	0.28 (0.15–0.5)
Fatigue	138	0.2 (0.17–0.24)	88	0.15 (0.12–0.19)	10	0.13 (0.07–0.24)
Nausea	184	0.27 (0.23–0.31)	68	0.12 (0.1–0.16)	8	0.12 (0.06–0.25)
Nasopharyngitis	116	0.73 (0.61–0.88)	42	0.3 (0.22–0.41)	8	0.4 (0.2–0.81)
Cardiac failure	126	1.84 (1.54–2.19)	62	1.14 (0.89–1.47)	6	0.89 (0.4–1.98)
Transient ischaemic attack*	268	9.56 (8.47–10.78)	47	2.42 (1.82–3.23)	5	2.5 (1.04–6)
Deafness*	148	6.72 (5.72–7.9)	20	1.09 (0.7–1.69)	5	2.24 (0.93–5.39)
Palpitations	40	0.4 (0.29–0.54)	20	0.26 (0.17–0.4)	5	0.56 (0.23–1.35)
Diarrhoea	73	0.13 (0.1–0.16)	35	0.07 (0.05–0.1)	4	0.06 (0.02–0.17)
Renal impairment	38	0.55 (0.4–0.75)	23	0.39 (0.26–0.58)	4	0.5 (0.19–1.32)
Dyspnoea	174	0.35 (0.31–0.41)	92	0.23 (0.19–0.28)	4	0.09 (0.03–0.23)
Hypersensitivity	100	0.62 (0.51–0.76)	56	0.42 (0.32–0.54)	4	0.26 (0.1–0.68)
Hypotension	43	0.25 (0.19–0.34)	29	0.21 (0.15–0.3)	3	0.18 (0.06–0.57)
Somnolence	12	0.07 (0.04–0.12)	14	0.1 (0.06–0.17)	3	0.2 (0.06–0.61)
Abortion Spontaneous	19	0.34 (0.19–0.6)	7	0.28 (0.13–0.58)	NR	NR

Abbreviation: The asterisk (*) indicates that this adverse reaction is not a common adverse reaction shared by all three drugs. PT, preferred term; ROR, reporting odds ratio; N, number of target adverse events of target drug; CI, confidence interval; NR, not report.

## 4 Discussion

This study is the first to apply the FAERS database to assess the safety of faricimab since its approval in 2022. We employed four disproportionality algorithms-ROR, PRR, MGPS, and BCPNN to evaluate AEs with faricimab as the PS drug. The ROR method is known for its sensitivity and ease of calculation, but it can generate false-positive signals when report volumes are low. PRR is a simple disproportionality measure designed to detect potential signals of adverse drug reactions. In contrast, BCPNN and MGPS offer higher specificity in identifying true signals (Wang et al., 2024b). BCPNN uses Bayesian methods to estimate the likelihood of a causal relationship between a drug and an adverse event, while MGPS adjusts effect sizes-such as PRR or ROR-to reduce the occurrence of false-positive signals (Wang et al., 2024a). Each of these algorithms has distinct advantages and limitations. Therefore, this study focused on discussing AEs that met the criteria of all four algorithms.

The global prevalence of w-AMD is projected to increase from approximately 196 million patients today to 288 million by 2040, similarly, the rising incidence of diabetes has led to a 64% increase in DR-related visual loss and a 27% increase in blindness (Keenan et al., 2021; Lin et al., 2021). Anti-VEGF therapies remain the mainstay for treating exudative retinal diseases, including n-AMD and DME. Faricimab, a bispecific antibody that binds with high affinity to both VEGF-A and Ang-2, was recently approved by the FDA (Sharma et al., 2020). The angiopoietin (Ang) yrosine kinase endothelial receptors (Tie) pathway plays a crucial role in the regulation of vascular homeostasis, vascular permeability, angiogenesis, and pro-inflammatory processes, notably, Tie-2 is a transmembrane receptor localized specifically on vascular endothelial cells, where it functions as a binding site for Ang-1 and Ang-2 (Ferro Desideri et al., 2023). Ang-1 serves as a complete agonist for Tie-2, promoting its phosphorylation and activating downstream signaling, which enhances vascular stability while suppressing vascular permeability and leakage. Conversely, Ang-2 functions as a partial agonist or antagonist of Tie-2. Consequently, Ang-2 binding to the Tie-2 receptor inhibits pathway activation, resulting in increased vascular leakage (Ferro Desideri et al., 2023). Under normal physiological conditions, Ang-1 is expressed at higher constitutive levels than Ang-2, however, under pathological conditions, Ang-2 expression is upregulated, amplifying the effects of VEGF through inhibition of the endothelial-specific Tie-2 receptor (Wolfrum et al., 2024). Faricimab’s dual mechanism of action demonstrated favorable outcomes in the YOSEMITE and RHINE trials, with improvements in baseline visual acuity, retinal structure, and extended treatment intervals maintained for up to 2 years (Wong et al., 2024). A meta-analysis by Watkins et al. (2023) found that Faricimab offers superior visual outcomes compared to Ranibizumab, with a safety profile similar to Aflibercept. The efficacy of faricimab is comparable to established first-line therapies, and the incidence of most adverse events is similar to that of existing alternative treatments (Zhang et al., 2024). However, Faricimab presents some potential adverse effects that warrant consideration. Based on our analysis, we highlight signals categorized as strong in adverse event reports for Faricimab, providing new insights into its safety in clinical applications.

The retina, along with the ciliary body, choroid, and iris, forms the posterior segment of the eye. Due to the low ocular bioavailability (less than 5%–10%) of topical medications, conventional treatments for posterior segment diseases often require peribulbar, retrobulbar, or subconjunctival injections, these procedures are invasive and may result in severe ocular complications (Puglia et al., 2021). Post-injection endophthalmitis is a rare yet severe complication of intravitreal anti-VEGF injections, potentially leading to significant vision loss (McCannel, 2011). In this study, diseases and symptoms associated with endophthalmitis showed strong signals across all four algorithms, consistent with the information provided in the drug label. The most common symptoms and signs of endophthalmitis include visual impairment, eye irritation, elevated intraocular pressure, vitreous floaters, eye pain, ocular hyperaemia, eyelid swelling, hypopyon, conjunctival congestion, corneal edema, and keratic precipitates (Haddock et al., 2014), each presenting strong signals as adverse reactions. Ben Ghezala et al. (2024). reported a case series of 6 patients with severe endophthalmitis, including 5 cases of severe anterior uveitis and intermediate uveitis resembling endophthalmitis. Several other clinical trials have also reported severe endophthalmitis associated with faricimab, suggesting a higher incidence of severe endophthalmitis with faricimab compared to first-generation anti-VEGF drugs (Thangamathesvaran et al., 2024; Chen et al., 2024; Palmieri et al., 2024). Endophthalmitis associated with faricimab administration may be attributed to several factors. Primarily, faricimab is supplied in a single-dose vial with a filter needle rather than in a prefilled syringe, which may increase the risk of contamination during handling (Alkhawaldeh and Abu Serhan, 2024). Secondly, thermal stability may contribute to these phenomena. The melting temperatures at which the heavy and light chains of faricimab unfold are approximately 64°C and 71.5°C, respectively, necessitating refrigeration (Akiba et al., 2019; Liberski et al., 2022). Additionally, faricimab’s anti-ANG-2 activity may also play a role, faricimab may possess pro-inflammatory properties, though this hypothesis is not yet fully supported by data (Ben Ghezala et al., 2024). Furthermore, cataract, retinal pigment epithelial tear, retinal vasculitis, retinal artery occlusion, and retinal vein occlusion all reached the positive thresholds in the four algorithms, aligning with the data presented in the drug label. Siddiqui et al. (2024) documented a case of faricimab-associated retinal vasculitis in a patient presenting with painless vision loss. Fluorescein angiography revealed delayed vascular filling with extensive leakage, characteristic of non-occlusive vasculitis, the patient’s symptoms improved following intravenous steroid treatment. Clemens et al. (2023) reported a case in which a patient developed a retinal pigment epithelial tear 4 weeks after transitioning from aflibercept to faricimab for intravitreal injections. In the phase three TENAYA and LUCERNE trials for nAMD, the incidence of retinal pigment epithelial tears was 2.7% and 3.0% in the faricimab group, compared to 1.8% and 0.9% in the aflibercept group (Heier et al., 2022). One possible explanation is that anti-VEGF therapy may induce fibrotic contraction of neovascular tissue beneath the retinal pigment epithelium, leading to a tear in the overlying epithelium and contributing to this observed complication (Ma et al., 2022). A single-center, prospective cohort study by Cancian et al. (2024). found that one patient with a history of ischemic heart disease died from acute myocardial infarction after 37 weeks of follow-up. Additionally, one patient developed retinal pigment epithelium changes following two injections, and another patient experienced two episodes of uveitis. A study by Mukai et al. (2024), conducted over the course of 1 year, found two cases of retinal pigment epithelium tear and one patient developed iritis after six intraocular injections of faricimab. The study by Mori et al. (2023) reported only allergic conjunctivitis associated with faricimab.

In addition to the AEs explicitly stated in the drug label, this study identified several unreported signals, including blindness, cerebral infarction, retinal hemorrhage, retinal occlusive vasculitis, glaucoma, dry eye, metamorphopsia, and unilateral blindness. While retinal occlusive vasculitis is rare, it remains a potential complication of faricimab treatment. Reichel et al. (2024) documented a case involving a 52-year-old male who developed sudden vision loss, new retinal hemorrhage, marked retinal vascular occlusion, and ischemia after receiving monthly faricimab injections for diabetic macular edema. In a separate case, a 72-year-old male receiving treatment for polypoidal choroidal vasculopathy developed occlusive vasculitis 2 weeks following his second faricimab injection (Chen et al., 2024). Faricimab and Brolucizumab may share similar mechanisms, with retinal occlusive vasculitis associated with both agents typically occurring between 1 week and 12 months after the first injection, rather than immediately post-injection (Monés et al., 2021). Anti-drug antibodies (ADAs) against a non-native form of Brolucizumab, formed upon prolonged incubation at body temperature in the vitreous, lead to immune complex formation, which mediates hypersensitivity reactions and triggers inflammation and platelet aggregation, this may be similar to faricimab (Reichel et al., 2024). Although the drug label for faricimab warns about the risk of thromboembolic events following its use, it does not explicitly mention any associated diseases. This study found that cerebral infarction is an adverse event that met the high-signal criteria across all four algorithms. Changes in blood flow, a hypercoagulable state, and vessel wall damage are three critical factors in the pathogenesis of thrombosis (Zhang et al., 2022). Intravitreal injection of anti-VEGF therapy may enter systemic circulation (Fogli et al., 2018). Following intravitreal administration, faricimab plasma concentrations increase in proportion to the dose, ranging from 0.5 to 3 mg, and reach peak plasma levels within 2 days. Faricimab is believed to undergo lysosomal degradation, resulting in peptides and amino acids similar to endogenous IgG (Panos et al., 2023). However, further research is needed to thoroughly understand the exact clearance rate, systemic absorption, and comprehensive pharmacokinetic profile of faricimab in humans. This study also found that glaucoma may be a potential adverse reaction to faricimab. A major risk factor for the development and progression of glaucoma is elevated intraocular pressure (IOP) (Zimmermann et al., 2021). Past experience suggested that repeated intravitreal injections of anti-VEGF drugs may reduce the function of the aqueous outflow system and be associated with the development of glaucoma (Wingard et al., 2019). A study by Wen et al. (2017) found that the aqueous outflow facility in eyes with AMD receiving 20 or more anti-VEGF injections decreased by 12% compared to the untreated fellow eye. Nitric oxide (NO) is a crucial signaling molecule. Anti-VEGF drugs disrupt the NO signaling pathway, potentially lowering NO levels below physiological baseline. This reduction in NO may contribute to glaucoma pathogenesis through mechanisms that lead to increased intraocular pressure (IOP), retinal vascular dysfunction, and retinal nerve fiber layer (RNFL) thinning (Daka et al., 2023). Endogenous VEGF expression in the trabecular meshwork functions as a paracrine regulator of conventional outflow pathways, Anti-VEGF drugs may disrupt this expression. Furthermore, several mechanisms, including inflammation, particle obstruction from injected solutions, or secondary angle-closure, could lead to increased IOP (Wen et al., 2017; Wen et al., 2016). A key characteristic of glaucoma is the death of retinal ganglion cells (RGCs). Multiple factors contribute to RGC damage, including oxidative stress, mitochondrial dysfunction, axonal transport blockade, synaptic impairment, glutamate-induced excitotoxicity, and alterations in pro-inflammatory cytokines, increasing evidence supports the role of TNF-α as a mediator of RGC death in glaucoma, acting through its binding to TNF receptor-1 (TNF-R1) (Romano et al., 2023). Prior to RGC death, there is typically a reversible phase of functional impairment and structural remodeling, non-IOP-dependent neuroprotection, or neuroprotection as an adjunct to IOP-lowering therapies for glaucoma, remains a significant challenge (Chou et al., 2018). RGC degeneration is commonly associated with ischemia resulting from central retinal artery occlusion and ischemic optic neuropathy. Gliosis, a pivotal event in the pathogenesis of glaucoma, serves as a hallmark of retinal degeneration. Reactive glial cells in the retina exhibit elevated immunoreactivity for glial fibrillary acidic protein (GFAP) and ionized calcium-binding adapter molecule 1 (Iba1), injury-induced gliosis in both the optic nerve head and retina accelerates retinal ganglion cell (RGC) death through the excessive release of pro-inflammatory mediators (Conti et al., 2021b; Conti et al., 2021a). A study by Amato R et al. found that diabetes can serve as an intraocular pressure (IOP)-independent risk factor for the early progression of glaucoma, contributing to oxidative stress and inflammation-induced RGC dysfunction, gliosis, and cell death (Amato et al., 2021). A significant proportion of patients treated with faricimab have diabetes, which itself increases the risk of developing glaucoma. In this study, retinal damage and changes associated with intravitreal injection of faricimab were observed, and glaucoma was also identified as a potential adverse reaction. Additionally, focusing on the protection of the optic nerve after treatment is crucial. Therefore, it is essential to monitor both the long-term and short-term IOP of patients after intravitreal injection of faricimab. In addition, clinicians should also be aware of the intraocular pressure increase and glaucoma induced by other intravitreal anti-VEGF drugs. Dry eye disease is a chronic inflammatory condition of the ocular surface (Kwaku Akowuah et al., 2024). Topical antibiotics are commonly used before and after intravitreal injection of anti-VEGF drugs, which may have toxic side effects on ocular surface cells. Additionally, most of these drugs contain preservatives (Laude et al., 2017).

Intravitreal injection of anti-VEGF drugs is widely used to mitigate disease progression and enhance visual outcomes in affected patients. Furthermore, it is important to compare the safety profiles of different anti-VEGF agents (Ventrice et al., 2013). Xiong et al. (2024) investigated ocular and systemic AEs following the market approval of brolucizumab through the FAERS database. They identified ocular events, including keratic precipitates (KPs), retinal perivascular sheathing, vitreal cells, dry eye, and glaucoma, as well as systemic events, such as arterial thromboembolic events, cerebral infarction, and rhinorrhea, which were not included in the drug’s prescribing information. Ma et al. (2022) analyzed three anti-VEGF drugs (ranibizumab, aflibercept, brolucizumab) using the FAERS database and identified several positive signals that were not mentioned in the drug’s prescribing information. These included macular ischemia and retinal pigment epithelial tear associated with ranibizumab, increased intraocular pressure and endophthalmitis associated with aflibercept, and retinal vasculitis and/or retinal vascular occlusion and dry eye associated with brolucizumab. Sakai et al. (2022) conducted a focused analysis of the relationship between intravitreal anti-VEGF injections and miscarriage using the JAPIC AERS and FAERS databases. They identified 19 miscarriage cases associated with ranibizumab, 6 with bevacizumab, and 4 with aflibercept. No cases of miscarriage associated with faricimab were observed in this study. However, considering that faricimab has been available for a shorter period and the sample size is smaller compared to other anti-VEGF drugs, this event should still be closely monitored. This study also compared the safety profiles of the three anti-VEGF drugs, identifying common adverse reactions. Furthermore, macular ischemia was found to be a potentially unique adverse reaction associated with ranibizumab, a finding consistent with the research of Ma et al. (2022) and others. Anti-VEGF drugs inhibit the normal physiological functions of VEGF. Consequently, VEGF blockade-induced vasoconstriction may exacerbate hypoxic damage in the already compromised macular capillary bed, potentially leading to detrimental effects on macular function and visual outcomes (Sun et al., 2021). Ranibizumab, which blocks all VEGF isoforms and has a Fab fragment that penetrates all retinal layers more effectively, exhibits a stronger effect (Ba et al., 2015). Additionally, while some ocular AEs were observed only in ranibizumab and aflibercept, the relatively short market availability of faricimab and its small sample size must be taken into account. Therefore, the results for this section should be interpreted with caution. Among the non-ocular AEs, myocardial infarction, transient ischemic attack, and deafness showed positive signals only in ranibizumab, cerebral infarction is a common side effect shared by the three anti-VEGF drugs. A recent study by Yang et al. (2025) found that the incidence of cardiovascular-related adverse events with Ranibizumab is higher than that with Aflibercept. This may be due to the drug’s impact on vascular tone (e.g., hypertension), rheological properties (e.g., promoting thrombosis), or cardiac electrophysiology, all of which increase the risk of cardiovascular events (Zakaria et al., 2022). Therefore, enhanced monitoring of the patient’s cardiovascular and cerebrovascular functions is recommended when administering anti-VEGF drugs via intravitreal injection.

This study has several limitations. First, the FAERS database presents a potential bias risk, as all reported information is voluntarily submitted by pharmaceutical companies, healthcare providers, and consumers. Although the majority of data in this study originates from physicians, a substantial portion is also provided by consumers, which may compromise data completeness and reliability. Furthermore, the FAERS database is the primary system in the United States for monitoring post-marketing adverse drug reactions. Approximately 55.6% of the data originates from the United States, the limited contributions from other regions undermine the external validity for other populations, ethnic group differences warrant consideration. Thus, cross-validation with other databases is recommended.

## 5 Conclusion

This study assessed the safety of intravitreal faricimab injections using the FAERS database, with data analyzed through four algorithms. Alongside confirming adverse reactions listed in the drug label, some previously unreported potential adverse reactions were also identified. Nevertheless, given the limitations of the FAERS database, these findings should be interpreted with caution. Future validation through rigorous prospective clinical trials or epidemiological studies is recommended.

## Data Availability

The original contributions presented in the study are included in the article/supplementary material, further inquiries can be directed to the corresponding author.
